# Olorofim for Invasive Fungal Diseases in Patients with Limited or No Treatment Options: A Systematic Review

**DOI:** 10.3390/pathogens15101041

**Published:** 2026-10-01

**Authors:** Felix Bratosin, Elena Hogea, Iulia Georgiana Bogdan, Horia Silviu Branea

**Affiliations:** 1 Transplant Infectious Diseases, Division of Infectious Diseases, McGovern Medical School at UTHealth Houston, Houston, TX 77030, USA; felix.bratosin@uth.tmc.edu; 2 Discipline of Microbiology, Multidisciplinary Research Center of Antimicrobial Resistance, Faculty of Medicine, “Victor Babes” University of Medicine and Pharmacy Timisoara, 300041 Timisoara, Romania; 3 Department of Infectious Diseases, “Victor Babes” University of Medicine and Pharmacy Timisoara, 300041 Timisoara, Romania; 4 Department of Internal Medicine I-Medical Semiotics II, “Victor Babes” University of Medicine and Pharmacy Timisoara, 300041 Timisoara, Romania; branea.horia@umft.ro

**Keywords:** antifungal agents, invasive fungal infections, aspergillosis, coccidioidomycosis, *Scedosporium*

## Abstract

Olorofim, the first orotomide antifungal, is active against *Aspergillus* spp., *Lomentospora prolificans*, *Scedosporium* spp. and *Coccidioides* spp. We reviewed published clinical reports of olorofim in invasive fungal disease (IFD) with limited or no treatment options. PubMed/MEDLINE, Europe PMC, Crossref, ClinicalTrials.gov and web sources were searched up to 1 September 2026 (PRISMA 2020; OSF registration). Trials, cohorts, case series and case reports of olorofim-treated patients were eligible; quality was appraised with JBI checklists and comparable safety and mortality data from the trial and the cohort studies were pooled (random-effects models), with case reports confined to narrative synthesis. Twenty-one reports were included: a single-arm phase 2b trial (202 evaluable patients) with three secondary reports, two cohorts (17 and 5 treated patients), a 3-patient series and 14 case reports. In the trial, adjudicated global response was 28.7% at day 42 and 27.2% at day 84 (75.2% and 63.4% including stable disease), with day-42 response of 42.3% in *Lomentospora* and 36.4% in *Scedosporium* infection; all-cause mortality was 11.9% and 16.3%; olorofim-attributed liver enzyme elevation occurred in 9.9%. Clinical success in disseminated coccidioidomycosis was 75.6% at day 42, with no deaths. Among 17 individually reported patients, infection was controlled in 10 and treatment failed in 5. Pooled olorofim-attributed liver enzyme elevation across non-overlapping trial and cohort groups was 10.8% (95% CI 4.0 to 26.3; I^2^ = 75%) and pooled early mortality was 19.5% (11.0 to 32.1; I^2^ = 44%). Adverse events were not addressed in 6 of the 14 case reports. In heavily pretreated patients, oral olorofim achieved strict global response in about one quarter, disease control in most and low early mortality; hepatotoxicity is the main safety signal, although safety reporting in the case literature is incomplete. Evidence is uncontrolled; randomised data are needed.

## 1. Introduction

Invasive fungal diseases (IFD) cause an estimated 3.8 million deaths each year, mostly in immunocompromised patients [1]. The World Health Organization fungal priority pathogens list ranks *Aspergillus fumigatus* as a critical-priority pathogen and includes *Scedosporium* spp., *Lomentospora prolificans* and *Fusarium* spp. among high-priority fungi because of their mortality and resistance to available drugs [2]. Only four systemic antifungal classes exist, each limited by toxicity, interactions, route of administration or spectrum [3]. Azole resistance in *A. fumigatus* is spreading, and *L. prolificans*, *Scedosporium* spp., *Fusarium* spp., *Microascus* spp. and *Rasamsonia* spp. are intrinsically resistant to most licenced agents, so that treatment often relies on combination regimens of uncertain benefit and on surgery [4,5].

Olorofim (F901318) is the first orotomide, a class that selectively inhibits fungal dihydroorotate dehydrogenase (DHODH) and blocks de novo pyrimidine synthesis [6]. It has no activity against *Candida* spp. or *Mucorales* but is highly active in vitro against *Aspergillus* spp., including azole-resistant and cryptic species, *Scedosporium* spp., *L. prolificans*, *Coccidioides* spp., *Microascus* spp., *Rasamsonia* spp. and other moulds, with minimum inhibitory concentrations (MICs) usually below 0.1 mg/L [7,8,9,10]. Efficacy has been shown in animal models of invasive aspergillosis, scedosporiosis, lomentosporiosis and central nervous system (CNS) coccidioidomycosis [11,12,13]. The drug is orally bioavailable, retains activity at sub-MIC exposures, distributes into the CNS and received Breakthrough Therapy designation from the United States Food and Drug Administration [14,15,16].

The first efficacy study, an open-label single-arm phase 2b trial (Study 32, NCT03583164), enrolled patients with proven IFD or probable invasive pulmonary aspergillosis who had failed, could not tolerate, or had no licenced option for their infection; its final results appeared in 2025 [17]. Secondary reports have since described the coccidioidomycosis subgroup, the effect of concomitant azoles, and paradoxical reactions during therapy [18,19,20]. Olorofim has also been used through compassionate-use and managed-access programmes, producing a French national cohort and case reports of *L. prolificans*, *Scedosporium*, *Microascus* and *Coccidioides* infections in children, transplant recipients and patients with primary immunodeficiency [21,22,23,24]. A randomised phase 3 trial in invasive aspergillosis (OASIS, NCT05101187) has released top-line results by press only [25]. Several questions remain open: the meaning of the trial’s strict composite end point in a salvage population [26]; whether the antagonism between azoles and olorofim seen in vitro and in mice matters clinically [27,28,29]; the frequency and management of hepatotoxicity [30,31]; and the risk of resistance selected by DHODH target mutations or by agricultural DHODH inhibitors [32,33]. No regulatory approval has yet been granted.

Existing reviews of olorofim are narrative and focus on pharmacology and preclinical data [7,16,34], and the only systematic reviews that mention the drug address in vitro activity of the antifungal pipeline against *Candida auris* or *Fusarium* spp. [35,36]. Most of the human evidence has appeared since 2023 and is scattered across a trial with complex end points, overlapping subgroup analyses, a compassionate-use cohort and case reports with outcomes ranging from cure to death. A systematic collation with explicit handling of overlapping populations, outcome definitions and safety reporting is therefore needed. The objective of this review was to identify, appraise and summarise all published clinical reports of olorofim treatment in humans, describing the populations, pathogens, regimens, clinical and mycological responses, mortality and adverse events, in patients of any age with IFD and limited or no alternative options.

## 2. Materials and Methods

### 2.1. Review Design, Protocol and Reporting Guidelines

This systematic review followed the Preferred Reporting Items for Systematic Reviews and Meta-Analyses (PRISMA) 2020 statement [37] and, for the narrative synthesis, the Synthesis Without Meta-analysis (SWiM) guideline [38]. The completed PRISMA 2020 checklist is provided in Supplementary Materials (File S1). The review protocol was registered with the Open Science Framework (OSF; registration code: osf.io/p9h4b). The review question, eligibility criteria, search sources, extraction items and appraisal tools were defined before screening. The full text of every included report was obtained and read. Screening, data extraction and quality appraisal were performed by a single reviewer (F.B.). Literature retrieval, data tabulation, statistical computation and drafting were supported by two reviewers (F.B. and I.G.B.), and all inclusion decisions, extracted values and analyses were re-checked by the authors against the source records. No ethical approval was required, and no external funding was received.

### 2.2. Eligibility Criteria

The population comprised patients of any age, sex or immune status with proven, probable or clinically diagnosed IFD of any type and site. The intervention was olorofim given systemically at any dose, for any duration, alone or with other antifungals, surgery or immunomodulation, within a trial or an access programme. No comparator was required. Outcomes were any clinical, radiological or mycological response, mortality, relapse, adverse events (particularly hepatotoxicity), treatment interruption, paradoxical reactions, drug interactions and plasma concentrations. Eligible designs were clinical trials, cohort studies, case series and case reports, including letters describing at least one patient, published in full in a peer-reviewed journal.

Reports were excluded if olorofim was not actually administered (for example, requested but unobtainable, tested only in vitro, or discussed as a future option), or if olorofim exposure was mentioned incidentally within a larger cohort without any olorofim-specific characteristics or outcomes. Conference abstracts, press releases, pharmacovigilance digests and preprints were excluded but catalogued. These sources were excluded because they have not undergone full peer review, rarely provide the detail required for extraction (confirmation that olorofim was actually given, numerators and denominators for response, and adverse-event information), cannot be appraised with the JBI checklists, and, for an investigational drug whose trial data are presented repeatedly at congresses before full publication, carry a high risk of duplicating patients already described in full reports. The catalogued records were nevertheless used in two ways: each was checked for a subsequent full publication, which was then screened in the usual way, and the remainder were compared with the included reports to judge whether clinically important evidence existed only in non-peer-reviewed form (Section 3.1). Reviews, editorials, commentaries, errata, in vitro, animal, pharmacokinetic modelling, cost-effectiveness and healthy-volunteer studies were excluded. No language, country or date limits were applied. When the same patients were reported more than once, all reports were included and their overlap was documented; no pooled count that would double count individuals was calculated.

### 2.3. Literature-Search Strategy

The literature search included publications available up to 1 September 2026. PubMed/MEDLINE was searched with the query “olorofim” and with “(F901318 OR orotomide) NOT olorofim” to capture reports published under the developmental code; together these returned 169 records. A publication-type search (“olorofim AND (systematic review OR meta-analysis)”) confirmed that no systematic review of the clinical question existed. Europe PMC and Crossref were queried with “olorofim” and “F901318”. ClinicalTrials.gov was searched for the interventions “olorofim OR F901318”, retrieving 22 registrations, of which two were treatment studies in patients (NCT03583164 and NCT05101187) and the remainder were phase 1 pharmacology studies. Embase, Web of Science and Scopus were not searched separately. Their omission was judged unlikely to affect the completeness of the review for three reasons: olorofim is a unique and unambiguous drug name with a single developmental code (F901318), so that retrieval does not depend on controlled vocabulary such as Emtree or on database-specific indexing, and a free-text search for these two terms performs equivalently in any bibliographic database; the clinical content of Embase, Web of Science and Scopus that is not already covered by MEDLINE consists mainly of conference abstracts and proceedings, which were excluded by design, and of journal articles that carry a digital object identifier and are therefore indexed by Crossref and, for the European biomedical literature, by Europe PMC, both of which were searched; and the database searches were supplemented by the structured web queries, full-text screening and citation searching described below, which target case reports that name olorofim only in the body of the article. Web of Science and Scopus would additionally have offered forward citation tracking; this function was covered by hand-searching the reference lists of the included studies and of the narrative reviews and by the web queries that combined olorofim with journal names.

Because olorofim is often named only in the body of case reports, the database searches were supplemented by more than fifty structured web queries combining “olorofim” with pathogen names, access programmes, sites of infection, outcome terms and journal or conference names (the complete list of the 52 query strings, grouped by term category, is given in Appendix A, Table A1), by screening of the Directory of Open Access Journals, Reactions Weekly digests and the sponsor’s press-release archive, and by full-text screening through PubMed Central of open-access case reports of *Lomentospora*, *Scedosporium*, *Microascus* and *Rasamsonia* infection and of coccidioidomycosis cohorts published from 2021 onwards. Reference lists of included studies and of narrative reviews were hand-searched. Full-text searching of Europe PMC, OpenAlex, Google Scholar and Semantic Scholar was attempted but was limited by access restrictions. Each web query took the form “olorofim” AND <term>, was run manually in Google Search and in Google Scholar, and was repeated on the closing date of the search (1 September 2026). For every query the first 50 results were screened by title and snippet, and any record that named olorofim in connection with a treated patient was retrieved in full and assessed against the eligibility criteria; records already retrieved by a previous query or by the database searches were logged as duplicates.

### 2.4. Study Selection and Data Extraction

**Figure 1:**
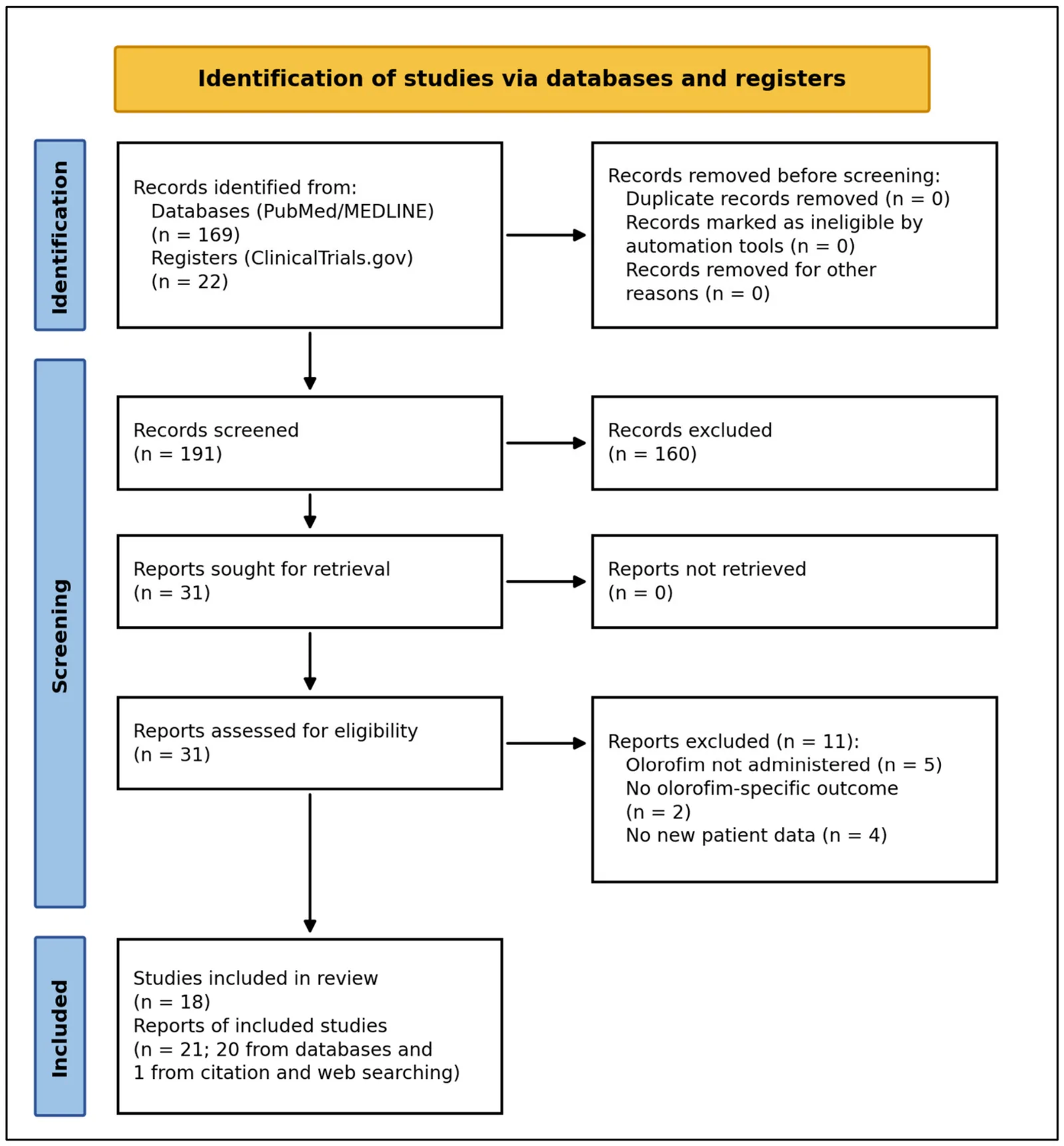
PRISMA 2020 flow diagram of study selection. Records identified from databases (PubMed/MEDLINE) and registers (ClinicalTrials.gov) are shown; one additional report identified through supplementary web and citation searching is included in the final count of 21 reports (18 studies).

PubMed records were screened at title and abstract level; records that were clearly in vitro, animal, modelling, review or resistance studies were excluded. All remaining records and all candidates from the supplementary searches were assessed in full. For each report, the reviewer first established whether olorofim had actually been given to at least one patient by reading the treatment narrative, and then whether any olorofim-specific characteristics or outcomes were reported. Reasons for exclusion were recorded, and the process is shown in a PRISMA 2020 flow diagram (Figure 1). Full texts were obtained from PubMed Central, publisher websites or institutional repositories.

Data were extracted into a structured form with fourteen domains: bibliographic details; full-text accessibility; confirmation of olorofim administration; design, setting, country, funding and registration; sample size, age, sex and underlying conditions; pathogen, site, EORTC/MSGERC category and olorofim MIC [39]; reason for use and prior antifungals; loading and maintenance dose, route, duration, drug monitoring, concomitant antifungals and surgery; outcome definitions and response rates with numerators and denominators; mortality; adverse events with attention to hepatic biochemistry, dose modification and discontinuation; follow-up; the authors’ conclusions; and verbatim quotations supporting each value. Values not reported were recorded as NR and were never estimated or inferred. Where a secondary report tabulated subgroup rows of the same trial, the rows were extracted as published. All values were re-verified against the source text before tabulation.

### 2.5. Quality Assessment and Evidence-Synthesis Methods

Quality was appraised with the Joanna Briggs Institute (JBI) checklists for quasi-experimental studies (the single-arm trial and its secondary analyses), case series (cohorts and multi-patient series) and case reports [40,41]. Items were rated as met, not met or unclear, and the item-level judgements are presented graphically with the number of items met; scores were not used to exclude studies. Risk of bias across the evidence was considered qualitatively, with attention to the absence of controls, non-random use of concomitant therapy, unblinded outcome assessment in case reports, selective publication and overlapping populations.

The primary synthesis was narrative, following SWiM: studies were grouped by design and pathogen, outcomes were summarised with the numerators and denominators reported by the authors, individual patients were classified as infection controlled, failed or not assessable according to the authors’ own description, and consistency across studies was examined [38]. Pooling of treatment response was judged inappropriate because the trial, the cohorts and the case reports used different response definitions and time points. Two outcomes were considered sufficiently comparable for an exploratory quantitative synthesis: liver enzyme elevation attributed to olorofim, and all-cause mortality within about three months of starting treatment. For these, proportions from non-overlapping patient groups drawn from structured study designs, namely the single-arm trial with its subgroup reports and the cohort studies, were combined with a random-effects model on the logit scale using the DerSimonian and Laird estimator, with exact binomial confidence intervals for individual groups and heterogeneity quantified by Cochran’s Q, I^2^ and τ^2^. Patients reported more than once were counted only once: secondary reports of the trial were entered as trial subgroups, the Faure series and the Michel report were regarded as part of the French cohort because they belong to the French programme, and the trial was entered as a whole for mortality because pathogen-specific mortality was reported only for aspergillosis and coccidioidomycosis. Single-patient case reports were confined to the narrative synthesis and were not entered into the pooled analyses, because case reports are subject to strong selective publication of exceptional responses and unexpected toxicities, lack a defined denominator and frequently omit adverse-event information. For transparency, the effect of additionally entering the 13 non-overlapping individually reported patients as a single aggregated group is reported as a sensitivity analysis. Where the azole-combination analysis of the trial reported event counts by stratum, unadjusted odds ratios with Woolf 95% confidence intervals were calculated to describe the association between azole co-administration and outcome; a continuity correction of 0.5 was applied to zero cells, and comparisons with no events in either stratum were not estimated. These comparisons are non-randomised and unadjusted. Publication bias was not assessed statistically because fewer than ten independent studies contributed to any outcome. Analyses were performed in Python 3.11 with NumPy and SciPy. The pooled proportions and the odds ratios were planned as exploratory, descriptive analyses: they summarise the available uncontrolled data and are not intended as precise estimates of event frequencies in future patients or as tests of hypotheses. Certainty of evidence was described qualitatively because comparative data do not exist.

## 3. Results

### 3.1. Study Selection

The study selection process is shown in Figure 1. The PubMed/MEDLINE searches retrieved 169 records and the ClinicalTrials.gov search 22 registrations. After screening, 160 records were excluded: 62 in vitro studies, 55 reviews, editorials or commentaries, 13 animal or pharmacokinetic studies, 8 records on unrelated topics, and 22 registry entries without patient outcome reports. Thirty-one reports were assessed in full, and 11 were excluded. In five, olorofim was named but not given to patients because it was unavailable; it was tested only in vitro or mentioned as a future option [5,42,43,44,45]. Two reports mentioned olorofim exposure of one patient without any olorofim-specific outcome [46,47]. Four contained no new patient data: a cost-effectiveness model, a drug-interaction analysis, a case commentary and an invited comment [26,48,49,50]. The coccidioidomycosis report was amended on 1 September 2026 to correct minor transcription errors, and the corrected version was used [18].

The supplementary web, registry and citation searches identified 16 further full-text reports that mentioned olorofim only in the body of the article. Fifteen were excluded because the drug was sought but not obtained, reported only as an MIC, or discussed without being given [51,52,53,54,55,56], or because two children in a coccidioidomycosis cohort received compassionate-use olorofim without any individual detail [57]. One case report of compassionate-use olorofim in a patient with cystic fibrosis was included [58]. Thirteen conference abstracts and the OASIS press release reported olorofim-treated patients but were excluded as abstract-only records [25,59,60,61]. These catalogued records were used to assess the completeness of the included evidence rather than as a source of data. Each abstract was checked for a subsequent full publication; abstracts reporting interim or subgroup analyses of the phase 2b trial [59,60] describe patients who are already covered by the full trial reports [17,18,19,20], and abstracts describing patients not identifiable in any full publication, such as four patients with refractory coccidioidal meningitis [61], did not provide the treatment detail, denominators or adverse-event information required for extraction and were noted as unpublished experience rather than as evidence. None of the catalogued records described a pathogen, indication or type of adverse event that was not represented among the included full reports, and none contradicted their findings; the sub-analyses still available only as abstracts are identified as a research priority in Section 4. The press release concerned the phase 3 OASIS trial, of which no full report had appeared by the search date [25].

In total, 21 reports of 18 studies were included: the phase 2b trial [17] and three secondary reports of it, namely the coccidioidomycosis subgroup [18], the azole-combination analysis [19] and a nested series of paradoxical reactions drawn from the trial and its managed-access programme [20]; a French compassionate-use cohort [21]; an Australian cohort of *Lomentospora* and *Scedosporium* rhino-sino-orbital and CNS infection in which five patients received olorofim [62]; a three-patient series of *Microascus* infection [22]; and 14 single-patient reports [23,24,30,31,58,63,64,65,66,67,68,69,70,71]. The three secondary reports re-analyse trial patients; the Faure series is re-reported within the French cohort, which states that four of its 17 patients had been published before; the adolescent described by Michel et al. was treated in the same national programme; and two case reports state that olorofim was given within a compassionate-use study or managed-access programme, the source of the 470-patient denominator used by Thompson et al. [20,31,71]. No pooled count of unique patients was therefore calculated. The non-overlapping evidence comprises 202 evaluable trial patients, 17 French cohort patients (including the Faure series), 5 Australian cohort patients and 13 further individually reported patients, about 237 patients in total.

### 3.2. Characteristics of the Included Studies

The characteristics of the 21 included reports are shown in Table 1.

The 21 reports were published between 2023 and 2026, 17 of them in 2025 or 2026 (Table 1). The only interventional study was the open-label single-arm phase 2b trial (Study 32), which enrolled 204 patients at 22 centres in 11 countries, treated 203 and analysed 202 after data review committee (DRC) confirmation of IFD; 124 were male and 78 female [17]. Three reports re-analysed these patients: the coccidioidomycosis subgroup of 41 patients from ten United States sites [18], the azole-combination analysis of all 202 patients [19] and the paradoxical-reaction series, whose six patients were drawn from 470 patients treated in the trial or in the managed-access programme that applied the same enrolment rules [20]. In the trial, 81 patients (40%) had high immunosuppression (haematological malignancy or stem-cell transplantation), 62 (31%) moderate immunosuppression (mainly solid-organ transplantation) and 59 (29%) low or no immunosuppression, of whom 39 had coccidioidomycosis; lung or sinonasal disease was present in 125 patients (62%), CNS disease in 48 (24%), bone or joint disease in 34 (17%) and disseminated disease in 50 (25%) [17]. In the coccidioidomycosis subgroup, 33 of 41 patients were male, 30 had CNS disease (13 with a shunt or reservoir) and 2 were immunosuppressed [18]. Of the six patients with paradoxical reactions, three were female, five came from the trial and four had no immunosuppression [20].

The French cohort identified 19 patients prescribed compassionate-use olorofim and analysed the 17 who received it: 14 were male, 7 were solid-organ transplant recipients (five lung), 4 had primary immunodeficiency (three chronic granulomatous disease, one CARD9 deficiency), 1 had received a haematopoietic stem-cell transplant, 3 had other conditions and 2 had no immunosuppression [21]. The Australian cohort comprised 18 onco-haematology patients (61% male, 53% with stem-cell transplantation) treated between 2010 and 2024; five received olorofim through the trial or compassionate access, because of progression in three, intolerance in one and subtherapeutic voriconazole concentrations in one [62]. The remaining 15 reports described 17 patients aged 8 to 73 years, 14 of them male; five were immunocompetent (traumatic inoculation or cystic fibrosis), six were transplant recipients and six had other conditions, including primary immunodeficiency, rheumatoid arthritis treated with a Janus kinase inhibitor, checkpoint-inhibitor neutropenia, unexplained lymphopenia and corticosteroid therapy [22,23,30,31,66,69]. Olorofim was accessed through the trial in four reports, through compassionate-use, expanded-access or managed-access programmes in the two cohorts and at least seven case reports, and through an emergency investigational new drug authorisation in one report [23,31,62,63,66]. No report included a comparative analysis; the Australian cohort contained 13 patients treated without olorofim and the Dutch report a second patient who did not receive the drug [58,62].

### 3.3. Pathogens, Indications and Treatment

Pathogens, sites of infection, reasons for olorofim use, susceptibility, dosing and co-interventions are shown in Table 2.

In the trial, *Aspergillus* spp. caused 101 of 202 infections (22 azole-resistant), followed by *Coccidioides* spp. (41), *L. prolificans* (26), *Scedosporium* spp. (22) and other fungi (12) [17]. In the French cohort, 23 strains were isolated from 17 patients: *A. fumigatus* in 8 (four of seven tested were azole-resistant, including one TR34/L98H strain), other *Aspergillus* species in 6, and *Scedosporium* and *Microascus* spp. in 3 each; five patients had mixed infection [21]. The 17 individually reported patients had infection with *L. prolificans* (7), *Scedosporium* spp. (4), *Microascus* spp. (3), *Coccidioides* spp. (2) or azole-resistant *A. fumigatus* (1), mostly at osteoarticular, ocular, endovascular or CNS sites [22,23,30,63,69]. Olorofim MICs were reported for 8 French patients, all 5 Australian patients and 7 individually reported patients and ranged from 0.007 to 0.5 mg/L; the same isolates usually had azole MICs of 8 mg/L or more and amphotericin B MICs of 4 mg/L or more [21,23,62,67,68,69].

Olorofim was given as salvage therapy in every report except one, in which it was added on day 7 of treatment for disseminated *L. prolificans* endocarditis [69]. In the trial, the reason for enrolment was failure of available therapy in 110 patients (54%), resistance to all licenced agents in 50 (25%) and intolerance in 29 (14%); 192 of 202 patients had received prior antifungal therapy (median two agents), and the median interval from diagnosis to olorofim was 75 days overall and 936 days in coccidioidomycosis, where patients had received a mean of 3.5 prior antifungals and 34 of 41 had received amphotericin B [17,18]. In the French cohort, 12 patients had refractory infection, 3 were intolerant of prior agents and 2 had no effective licenced drug; all had received prior antifungals (median two regimens over 9.1 months) and 16 had received an azole [21]. The first 58 trial patients received a weight-based regimen (180 to 300 mg on day 1, then 120 to 240 mg per day); later trial patients and almost all compassionate-use patients received 150 mg twice on day 1 and then 90 mg twice daily [17,21]. In the trial, 60 mg twice daily was used with strong CYP3A or CYP2C9 inhibitors and escalation to 120 and 150 mg twice daily was permitted; population pharmacokinetic modelling indicated target exposure in 99% of patients [17,18]. Other regimens were a higher loading dose in two *Microascus* cases, a reduced dose during voriconazole co-administration, weight-based dosing in two children (30 mg twice daily in a 26 kg child, troughs 0.51 and 0.53 mg/L; 3 mg/kg per day in a 14-year-old, troughs 1.24 and 1.22 mg/L) and escalation to 150 mg twice daily during monotherapy for endocarditis [22,23,63,64,69]. A trough of 0.586 mg/L, 39 times the MIC, was measured in the adult with *Scedosporium* arthritis [68]; no other report described drug monitoring.

The trial’s main phase lasted a median of 84 days; 114 of 203 patients entered an extended phase with a median total duration of 309 days (range 38 to 988), 49 were treated for more than one year, and in coccidioidomycosis the median duration was 409 days and 38 of 41 patients entered the extended phase [17,18]. French responders were treated for a median of 9 months, and patients still on therapy at the end of follow-up for a median of 26.3 months [21]. Individual courses ranged from 3 days to 11 months, and 6 months was the usual planned duration for osteoarticular infection [23,66,70,71]. In the trial, 125 of 202 patients received olorofim essentially as monotherapy; a second potentially effective agent for 15 days or more was given to 31 of 41 coccidioidomycosis patients and 21 of 161 others, and an azole for at least two days to 64 of 202 [17,19]. Combination therapy was used in 14 of 17 French patients, 1 of 5 Australian patients and, at some point, 8 of the 17 individually reported patients [21,24,62,69]. Surgery accompanied olorofim in all five osteoarticular cases and in the endocarditis, endophthalmitis and redo lung-transplant cases [24,65,67,69].

### 3.4. Clinical Outcomes and Safety

Efficacy, mortality, adverse events and follow-up are summarised in Table 3.

In the trial, the DRC-adjudicated global response, which required at least partial improvement in clinical, radiological and mycological components, was achieved by 58 of 202 patients (28.7%, 95% CI 22.6 to 35.5) at day 42 and 55 of 202 (27.2%, 95% CI 21.2 to 33.9) at day 84; with stable disease counted as success the rates were 75.2% and 63.4%, and clinical response was 59.9% and 54.0% [17]. All-cause mortality was 11.9% at day 42 and 16.3% at day 84; no death was judged treatment-related, and all 33 deaths by day 84 occurred in patients with high or moderate immunosuppression, 26 of them with aspergillosis [17]. Day-42 global response was 34.7% in aspergillosis, 42.3% in *L. prolificans* infection, 36.4% in *Scedosporium* infection and 33.3% in other fungi, and day-84 mortality was 25.7%, 11.5%, 13.6% and 8.3%, respectively [17]. Among the 101 aspergillosis patients, day-42 global response was 3 of 9 with a mould-active azole, 8 of 15 with fluconazole and 24 of 77 without an azole, and day-84 mortality was 3 of 9, 4 of 15 and 19 of 77 [19]. In coccidioidomycosis, no patient met the global success definition, whose serological component could not be met within 84 days; stable disease was adjudicated in 39 of 41 at day 42, clinical success was 75.6% at day 42 and 73.2% at day 84 with no deaths, and day-42 clinical success was 26 of 30 with and 5 of 11 without a concomitant azole [18,19]. During extended treatment, investigator-assessed clinical success in coccidioidomycosis was 26 of 38 (68.4%) at day 180 and 24 of 39 (61.5%) at the last visit (median day 430), with four deaths, three from coccidioidomycosis; complement fixation titres did not fall fourfold in any of the 12 patients with serology after more than one year of treatment [18]. Across the whole trial, 83 of 114 patients completed extended treatment, investigator-assessed success at the last observation was 46% for global and 79% for clinical response, and 50 of 203 patients (24.6%) died during the entire study [17].

In the French cohort, 5 of 15 patients treated for at least 15 days achieved complete success, allowing discontinuation after a median of 9 months, 7 had a partial response with ongoing therapy and 3 died with refractory infection; 3-month mortality was 5 of 17, including two deaths within 15 days of starting the drug, and one patient relapsed 5 months after stopping and responded to resumption [21]. All three *Microascus* and two of three *Scedosporium* infections achieved complete success, most *Aspergillus* infections achieved partial response, and the single *L. prolificans* infection failed [21]. In the Australian cohort, four of the five olorofim recipients were alive at day 30 and three beyond day 180, compared with 30-day and 180-day case-fatality rates of 56% and 67% in the cohort as a whole [62]. Among the 17 individually reported patients, infection control was described in 10: the three *Microascus* cases, three of five osteoarticular *L. prolificans* or *Scedosporium* infections treated with surgery, disseminated *L. prolificans* abscesses that resolved on PET, the cystic fibrosis patient whose haemoptysis ceased, the redo lung-transplant recipient and the adolescent with chronic granulomatous disease and TR34/L98H *A. fumigatus* infection, who remained in complete remission on MRI after two years of olorofim following lobectomy [22,23,31,58,63,64,65,66]. Five patients failed: two with disseminated *L. prolificans* endophthalmitis or endocarditis died after 116 and 76 days, one elbow osteomyelitis required amputation, one *Scedosporium* arthritis persisted despite a trough 39 times the MIC, and one coccidioidal meningitis progressed after 11 months [24,67,68,69,71]. Two patients were not assessable: one treated for three days before death and one whose report addressed hepatotoxicity only [30,70]. Paradoxical worsening occurred in 6 of 470 olorofim-exposed patients (1.3%), three with coccidioidomycosis, two with *Scedosporium* infection and one with *L. prolificans* sinusitis, at a median of 41 days (range 6 to 253) after starting therapy; within the trial the rate was 3 of 59 in patients without immunosuppression, 1 of 62 with moderate and 1 of 81 with high immunosuppression. All reactions resolved within 7 to 87 days, olorofim was continued in all (one brief pause), and all six patients subsequently responded [20].

In the trial, 45 of 203 patients (22.2%) had a grade 3 hepatic adverse event of special interest, of which 20 (9.9%) were judged at least possibly related to olorofim by an independent hepatic advisory committee; five were serious, 14 resolved after dose modification, 6 (3.0%) led to permanent discontinuation and none was fatal. Most hepatic events began within three months, with a peak between two and six weeks, and neither prior azole-related liver injury nor olorofim exposure predicted them [17]. Gastrointestinal events occurred in 107 patients (53%) and were judged related in 20 (9.9%); ciclosporin and tacrolimus doses were reduced in 9 of 18 and 8 of 46 evaluable patients [17]. Olorofim-attributed hepatic events occurred in 9 of 41 coccidioidomycosis patients (22.0%), all within three months, with one discontinuation on day 109, and in 2 of 34 versus 9 of 127 non-coccidioidal patients with and without azole co-administration [18,19]. In the French cohort, one patient had an alanine aminotransferase (ALT) elevation of three times the upper limit of normal that resolved with dose adjustment, one interrupted therapy temporarily for nausea attributed to voriconazole and one transplant recipient required a tacrolimus dose increase; no severe related event occurred [21]. In the individual reports, hepatotoxicity interrupted therapy in three patients: probable hepatocellular drug-induced liver injury (peak ALT 1642 U/L) at four to five weeks, which resolved and did not recur at 30 mg twice daily; transaminitis (ALT 1630 U/L) after four months that recurred on rechallenge and led to permanent cessation; and hepatitis attributed to an interaction with high-dose fluconazole that resolved when fluconazole was stopped [30,31,71]. Raised tacrolimus and everolimus troughs were noted in one lung-transplant recipient [22], and no adverse event was observed during two years of olorofim in the adolescent with chronic granulomatous disease [63]. The absence of olorofim-related adverse events was stated in seven reports, whereas adverse events were not addressed at all in six of the 14 single-patient reports. Safety information from the case literature is therefore incomplete: the three hepatotoxicity events described above represent a minimum count, the absence of a reported adverse event in a case report cannot be equated with the absence of an event, and the published case literature is subject to safety reporting bias, because unexpected toxicities are more likely to be written up than an uneventful course. Follow-up ranged from 3 days to more than 3 years and was not stated in four reports.

### 3.5. Quantitative Synthesis

**Figure 2:**
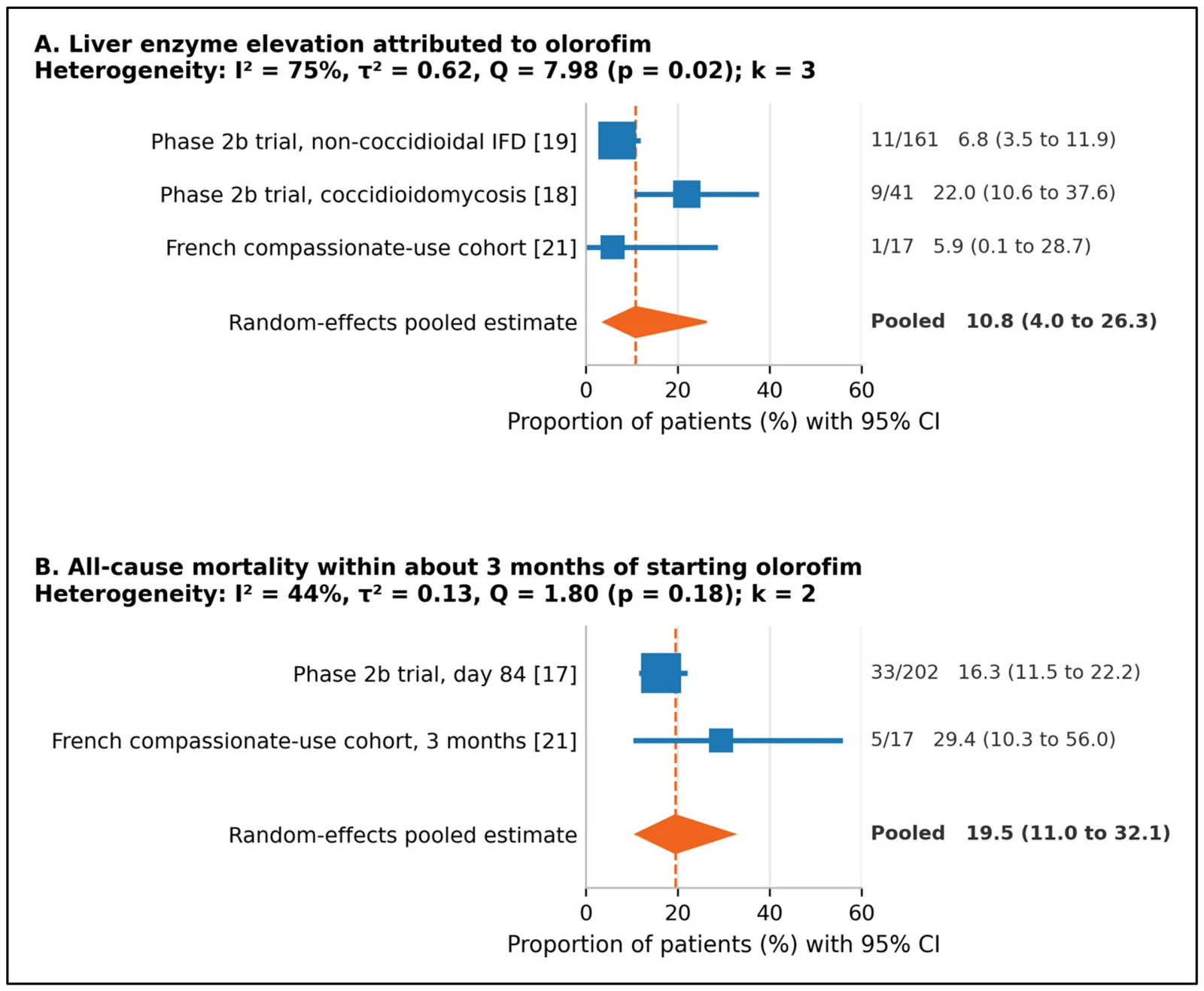
Random-effects pooled proportions (logit transformation, DerSimonian and Laird estimator) of (**A**) liver enzyme elevation attributed to olorofim and (**B**) all-cause mortality within about three months of starting olorofim, across non-overlapping patient groups from the phase 2b trial and the French compassionate-use cohort; single-patient case reports were not pooled. Squares show group proportions with exact 95% confidence intervals (CI), sized by group size; diamonds show the pooled estimate. The non-coccidioidal trial group is the sum of the two azole strata reported in the azole-combination analysis [19]; the coccidioidomycosis group is the trial subgroup [18]; the trial mortality is the day-84 value [17]; the French cohort is reported at three months [21]. IFD, invasive fungal disease.

Treatment response was not pooled because the trial, the cohorts and the case reports used different response definitions and time points. Two outcomes were combined across non-overlapping patient groups from the trial and the cohort studies (Figure 2); single-patient case reports were confined to the narrative synthesis above and were not pooled. For olorofim-attributed liver enzyme elevation, three groups were entered: the non-coccidioidal trial patients (11 of 161, the sum of the two azole strata reported in the azole-combination analysis) [19], the coccidioidomycosis trial subgroup (9 of 41) [18] and the French cohort (1 of 17) [21]. The random-effects pooled proportion was 10.8% (95% CI 4.0 to 26.3), with substantial heterogeneity (I^2^ = 75%, τ^2^ = 0.62, Q = 7.98, *p* = 0.02). For all-cause mortality within about three months, two groups were entered: the trial at day 84 (33 of 202) [17] and the French cohort at 3 months (5 of 17) [21]; the Australian cohort was not entered because mortality among olorofim recipients was reported only at day 30 and beyond day 180 [62]. The pooled proportion was 19.5% (95% CI 11.0 to 32.1), with moderate heterogeneity (I^2^ = 44%, τ^2^ = 0.13, Q = 1.80, *p* = 0.18), although heterogeneity statistics derived from two groups are imprecise. The trial contributed most of the weight in both analyses (42% and 40% for its two groups in the hepatic analysis and 72% in the mortality analysis). In a sensitivity analysis that additionally entered the 13 individually reported patients who do not overlap with the French cohort or the trial as a single aggregated group (olorofim-attributed liver enzyme elevation in 3 of 13 [30,31,71]; death within 120 days of starting olorofim in 3 of 13 [24,69,70]), the pooled proportions were 13.0% (95% CI 5.7 to 26.7; I^2^ = 69%) for liver enzyme elevation and 18.0% (95% CI 13.3 to 24.0; I^2^ = 2%) for mortality. These values are given for transparency only, because the aggregated case-report group has no defined denominator and is subject to selective publication and incomplete safety reporting.

**Figure 3:**
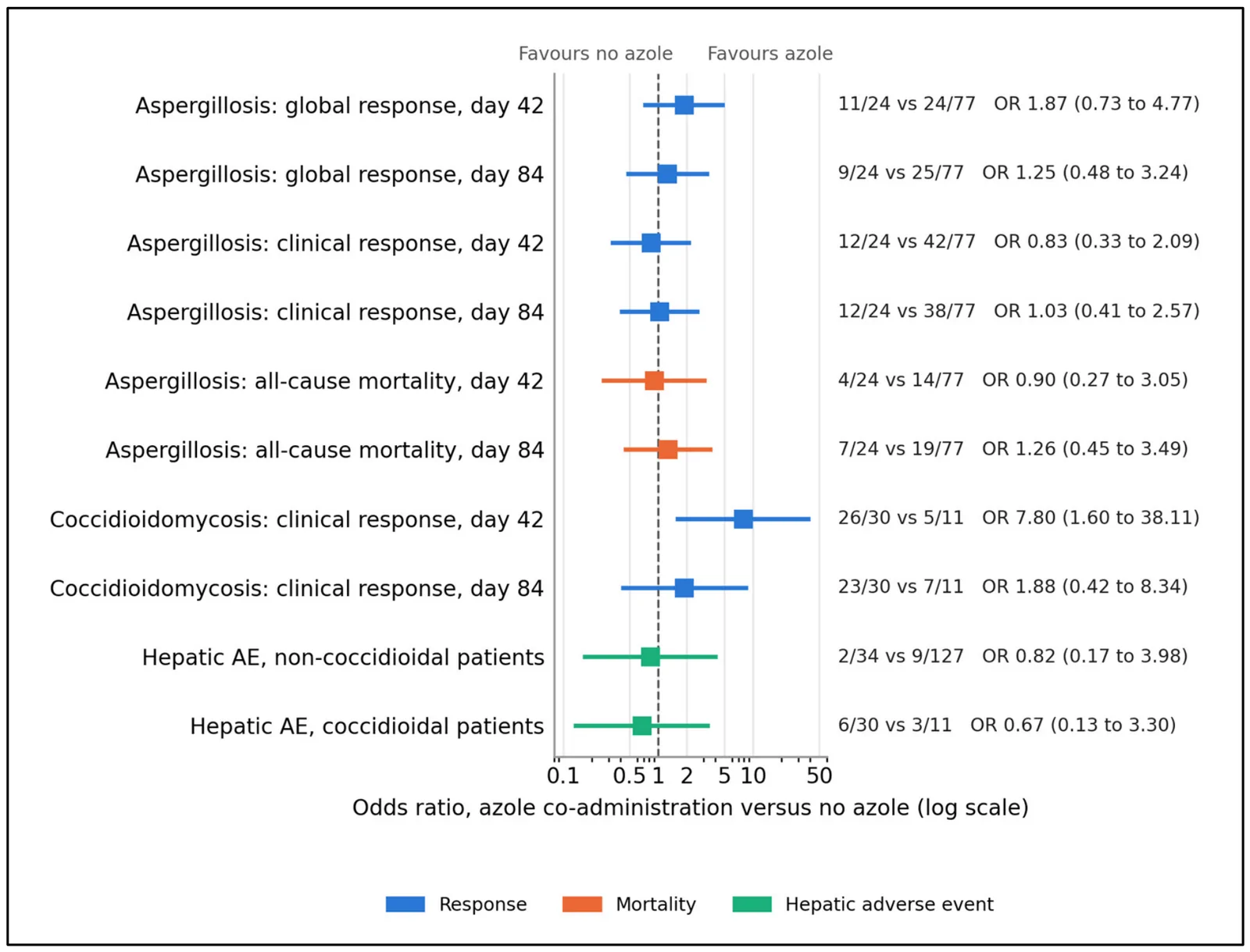
Reviewer-calculated unadjusted odds ratios (OR) with 95% confidence intervals for azole co-administration (at least two days) versus no azole in the phase 2b trial, by pathogen group and end point, using the counts published in the azole-combination analysis [19]. Allocation to azole co-administration was not randomised. Global and clinical responses were adjudicated by the data review committee at day 42 and day 84. Hepatic AE, liver enzyme elevation at least possibly attributed to olorofim. Mortality in coccidioidomycosis (0/30 versus 0/11) cannot be estimated because both groups had zero events.

Reviewer-calculated unadjusted odds ratios (OR) for azole co-administration for at least two days versus no azole in the trial, by pathogen group and end point, are shown in Figure 3 [19]. In aspergillosis, the OR for global response was 1.87 (95% CI 0.73 to 4.77) at day 42 and 1.25 (0.48 to 3.24) at day 84, for clinical response 0.83 (0.33 to 2.09) and 1.03 (0.41 to 2.57), and for death 0.90 (0.27 to 3.05) and 1.26 (0.45 to 3.49). In coccidioidomycosis, the OR for clinical response was 7.80 (1.60 to 38.11) at day 42 (26 of 30 versus 5 of 11) and 1.88 (0.42 to 8.34) at day 84 (23 of 30 versus 7 of 11); no death occurred in either stratum. The OR for olorofim-attributed liver enzyme elevation was 0.82 (0.17 to 3.98) in non-coccidioidal patients (2 of 34 versus 9 of 127) and 0.67 (0.13 to 3.30) in coccidioidal patients (6 of 30 versus 3 of 11).

Publication bias was not assessed statistically because fewer than ten independent studies contributed to any outcome, the trial accounted for most of the weight in each synthesis and, after restriction of the pooled analyses to trial and cohort data, the only other contributing study was the French cohort.

### 3.6. Quality Assessment

**Figure 4:**
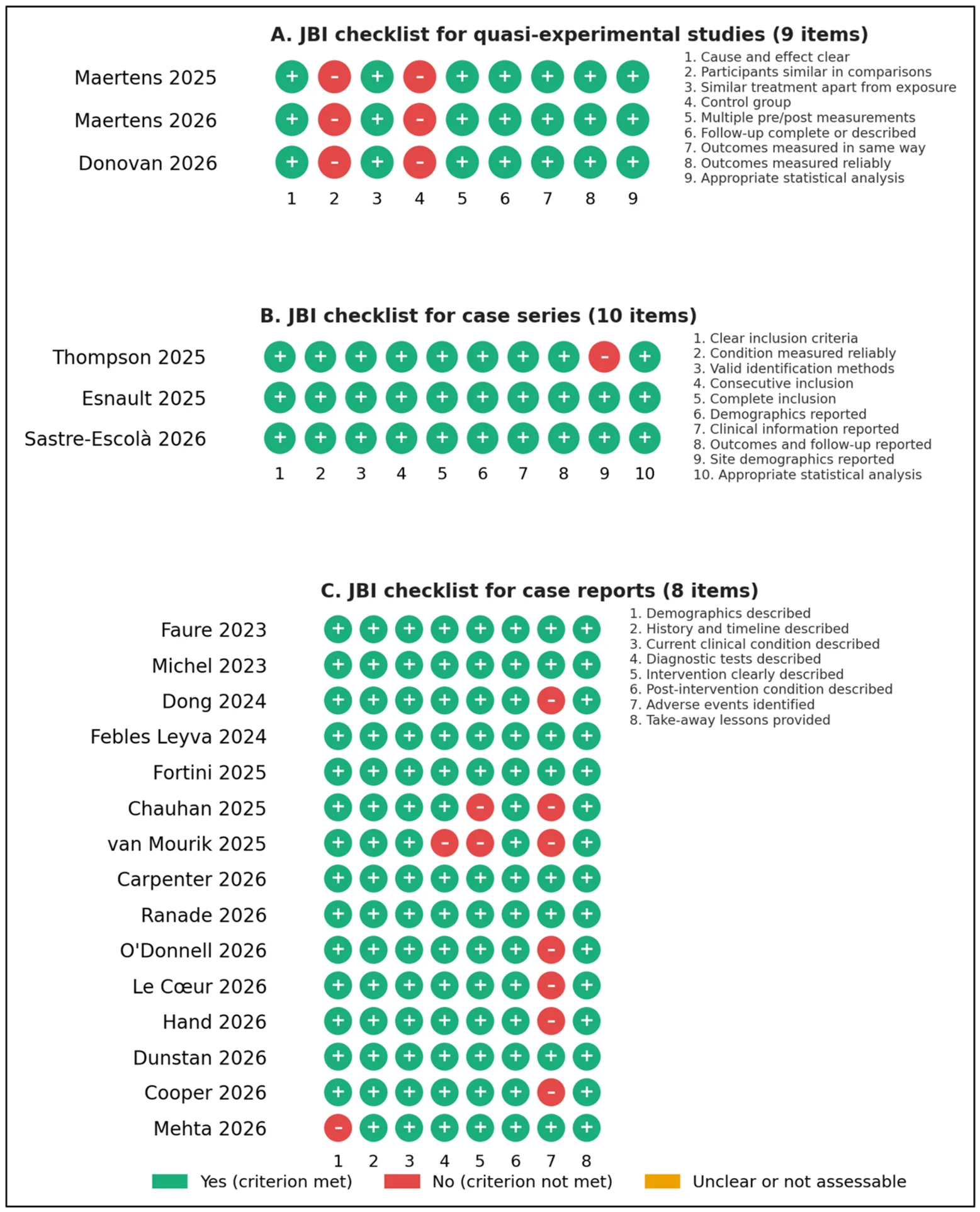
Methodological quality of the included reports appraised with the Joanna Briggs Institute (JBI) critical appraisal checklists for (**A**) quasi-experimental studies, (**B**) case series and (**C**) case reports [40,41]. Green, criterion met; red, criterion not met; amber, unclear. Reports assessed: [17,18,19,20,21,22,23,24,30,31,58,62,63,64,65,66,67,68,69,70,71].

The item-level appraisal is shown in Figure 4. The trial and its two subgroup reports met seven of nine JBI criteria for quasi-experimental studies; the unmet items were the absence of a control group and, in the sub-analyses, the non-random assignment of concomitant azoles with small subgroups [17,18,19]. The paradoxical-reaction series met nine of ten case-series criteria, with site demographics not reported [20]; the French and Australian cohorts met all ten, with retrospective and unblinded outcome assessment in both [21,62]. Seven reports, including the *Microascus* series, met all eight case-report criteria [22,23,30,31,63,64,66]; six met seven, usually because adverse events were not addressed [24,67,68,69,70,71]; one met six because neither the olorofim dose nor adverse events were reported [65]; and one met five [58].

## 4. Discussion

This review collated the published clinical experience with olorofim. The evidence consists of one single-arm phase 2b trial with three secondary reports, two retrospective cohorts, one three-patient series and 14 single-patient reports, covering about 237 patients with extractable data and an overlapping managed-access population of 470 patients. The main findings are consistent across designs. In heavily pretreated patients with proven IFD or probable invasive pulmonary aspergillosis and few or no alternatives, olorofim produced a strict adjudicated global response in 28.7% of patients at day 42 and stabilisation or better in 75.2%, with day-42 and day-84 all-cause mortality of 11.9% and 16.3% [17]. In disseminated coccidioidomycosis, three quarters of patients achieved adjudicated clinical success with no deaths within 84 days [18]. In the French compassionate-use cohort, one third of evaluable patients achieved complete success and four fifths achieved at least partial control, at the cost of a 29.4% three-month mortality that the authors attributed largely to the severity of the underlying conditions [21]. Among individually reported patients, infection control was described in ten of 17, most convincingly in *Microascus* bronchopulmonary infection and in osteoarticular or soft-tissue *L. prolificans* and *Scedosporium* infection where extensive surgical source control was possible, whereas disseminated, endovascular and CNS *L. prolificans* disease and refractory coccidioidal meningitis frequently failed [22,23,66,69,71]. The safety profile was consistent: hepatic enzyme elevation was the main safety signal, occurred in roughly 10% of trial patients and about 20% of those with coccidioidomycosis, was generally reversible with dose modification, but was severe enough to require permanent cessation in a small minority, and gastrointestinal intolerance was common but mild [17,18,30,31]. The exploratory pooled estimates from the trial and cohort data, 10.8% for olorofim-attributed liver enzyme elevation and 19.5% for mortality within about three months, are consistent with these study-level values; the overlapping confidence intervals of the trial and the French cohort for mortality suggest that early survival in compassionate use resembles that in the trial, although only two groups contributed to this estimate and the case reports were deliberately excluded from it, whereas the substantial heterogeneity of the hepatotoxicity estimate reflects a real difference between coccidioidomycosis and other infections rather than random variation. Both estimates are dominated by the trial and should be read as descriptive summaries of the available data rather than as precise population values.

Comparisons between studies must allow for differences in populations, outcome definitions and time horizons. The pathogens treated mirror the in vitro spectrum of olorofim, and dosing was nearly uniform, so differences in outcome are more plausibly related to pathogen, site and host than to the regimen. The trial’s strict primary end point counts stable disease as failure. This is demanding in a salvage population in which many patients had chronic osteoarticular, CNS or cavitary disease that cannot improve radiologically within six weeks. The 75.2% rate including stable disease and the 59.9% clinical response rate are closer to what clinicians and the French investigators describe as control of infection, a point also made in the accompanying commentary [17,21,26]. The French cohort corroborates the trial: its success rate of 33.3% is numerically similar to the trial’s global response despite a different definition, a longer treatment duration (median 9 months in responders) and near-universal combination therapy, which suggests that the trial result is broadly reproducible outside a protocol. Pathogen-specific comparisons are limited: aspergillosis outcomes in the trial (day-84 global response 32.5% and mortality 24.7% without azoles) were less favourable than coccidioidomycosis outcomes (clinical success 73.2%, mortality 0%), reflecting the very different host populations, since 87.6% of non-coccidioidal patients but only 4.9% of coccidioidal patients were substantially immunosuppressed [18,19]. The case-report literature, dominated by *L. prolificans* and *Scedosporium* spp., can now be set against the trial’s pathogen-specific results: day-42 global response was 42.3% in *L. prolificans* and 36.4% in *Scedosporium* infection, and day-84 mortality was 11.5% and 13.6%, lower than in aspergillosis (25.7%), which the trial authors linked to the predominance of immunocompetent, post-traumatic hosts among these infections [17]. The individually reported *L. prolificans* patients had a worse course, with three deaths among seven, but they were selected for disseminated, endovascular or CNS disease, whereas the trial’s *Lomentospora* group was mostly osteoarticular or pulmonary; the authors of the two fatal disseminated cases considered survival of 116 and 76 days longer than the historical median of 6 to 9 days for such disease [17,24,69,70]. The Australian cohort adds that three of five olorofim recipients survived beyond 180 days in a population whose overall 180-day case fatality was 67%, although the authors attributed part of this difference to survivorship, because olorofim was obtained only by patients who had survived initial therapy [62]. An interim analysis cited in one of the excluded laboratory reports is superseded by these final figures [47]. Concomitant antifungals were common despite the preclinical antagonism data, and the findings on azole co-administration showed no clear clinical antagonism: the trial authors concluded that aspergillosis outcomes were similar or numerically favoured olorofim without a mould-active azole, none of the reviewer-calculated odds ratios for response, mortality or hepatotoxicity in aspergillosis excluded the null, and the only estimate that did, a higher day-42 clinical response with azoles in coccidioidomycosis, is best explained by confounding by indication, because azoles were continued at the discretion of the treating physician, patients who could continue an azole were probably less ill, and the difference was not sustained at day 84. The sub-analysis was non-randomised, the mould-active azole group in aspergillosis contained only nine patients, and one case report described deterioration on olorofim monotherapy with improvement after voriconazole and terbinafine were re-added, so the question remains open [19,24,27,29].

The clinical implications are cautious. Olorofim is the only oral agent with reproducible activity against *L. prolificans* and *Scedosporium* spp., organisms for which the current standard of voriconazole with terbinafine is associated with mortality above 50% in registry data and in the Australian cohort included here [4,5,62]. The included reports indicate that olorofim can achieve durable cure of localised osteoarticular and soft-tissue infection by these organisms when combined with aggressive surgery, and can control refractory *Microascus*, azole-resistant *Aspergillus* and disseminated coccidioidomycosis in a substantial proportion of patients, which supports its use in these settings when accessible through trials or named-patient programmes [18,22,23,66]. Because surgical source control accompanied olorofim in all osteoarticular cases and in the endocarditis, endophthalmitis and redo lung-transplant cases, the contribution of the drug alone cannot be isolated in these patients [24,65,67,69]. At the same time, the reports of failure in disseminated *L. prolificans* endocarditis and meningitis, the failure of prolonged monotherapy in a *Scedosporium* arthritis despite plasma exposure far above the MIC, the need for amputation in an elbow osteomyelitis and the late failure in coccidioidal meningitis caution against expecting olorofim alone to overcome high fungal burdens, poorly vascularised bone or cerebral disease, and highlight the sparse data on drug penetration into bone and joints [15,67,68,69,71]. Clinicians should anticipate hepatotoxicity, obtain baseline liver biochemistry, monitor at least every two weeks during the first months as recommended by the authors of the drug-induced liver injury report, and consider dose reduction rather than automatic cessation because several patients tolerated a reduced dose [17,21,30]. Interactions with calcineurin and mTOR inhibitors and with high-dose fluconazole should be expected, and paradoxical worsening within the first weeks to months should be recognised as a possible immune reconstitution-like phenomenon rather than treatment failure [20,22,71]. The long treatment durations reported, often exceeding a year, also have implications for adherence, cost and monitoring, and the cost-effectiveness model published for invasive aspergillosis in patients with limited options will need to be updated as new data emerge [48].

Heterogeneity is intrinsic to this evidence base and explains the conflicting findings. Responses varied with pathogen and site more than with the drug regimen, which was nearly uniform; the clearest gradient runs from localised *Microascus*, *Scedosporium* and *L. prolificans* infections amenable to surgery, where most patients were cured, to disseminated or CNS *L. prolificans* disease, where all reported patients died, and to coccidioidal meningitis, where one adjudicated trial subgroup did well while both individually reported patients failed or could not be assessed [21,24,30,69,70,71]. Publication bias could not be tested because too few independent studies exist, but it plausibly operates in both directions. This is because dramatic cures and instructive failures are more likely to be written up than unremarkable partial responses, and the near-exclusive use of olorofim as late salvage therapy, often after months of ineffective treatment or, in one case, three days before death, systematically disadvantages the drug. For this reason, single-patient reports were confined to the narrative synthesis and excluded from the pooled analyses, and the true frequency of toxicity in compassionate use remains unknown because adverse events were not addressed in six of the 14 single-patient reports, a safety reporting bias that makes the published case literature an unreliable source of adverse-event frequencies and means that the toxicities it describes should be regarded as a minimum. Future research should prioritise the peer-reviewed publication of the phase 3 OASIS trial, whose press-released top-line results reported non-inferior day-42 all-cause mortality for olorofim compared with liposomal amphotericin B followed by standard of care in 225 randomised patients with invasive aspergillosis, together with fewer drug-related adverse events [25]; publication of the CNS and paediatric sub-analyses of Study 32 presented so far only as abstracts [60]; the randomised placebo-controlled COCCIMETRICS study in disseminated coccidioidomycosis (NCT07712484); prospective international registries of compassionate-use patients with standardised EORTC/MSGERC outcome assessment and mandatory adverse-event reporting; pharmacokinetic studies of bone, joint, ocular and CSF penetration; therapeutic drug monitoring studies to define exposure-toxicity relationships for hepatotoxicity; and surveillance for DHODH-mediated resistance in clinical isolates, given the demonstrated cross-resistance with agricultural DHODH inhibitors [32,33].

### Limitations

This review has several limitations. The evidence contains no controlled study, so no comparative effect of olorofim can be estimated, and most reports are single cases with unblinded, investigator-assessed outcomes, variable follow-up and, in six of the 14 single-patient reports, no information on adverse events, so safety reporting bias within the case literature is likely and case reports contribute to the description of toxicity only as a minimum count. Overlap between the trial, its secondary reports, the managed-access programme, the French cohort and several case reports prevents a reliable count of unique patients, and the main risks of bias across the evidence are the absence of any comparator, selective publication, unblinded outcome assessment, overlapping populations and incomplete safety reporting. The pooled proportions are exploratory: they combine only two or three groups from the trial and the French cohort, so that heterogeneity statistics are imprecise, use approximately matched time points for mortality and are dominated by the trial, and the odds ratios for azole co-administration are unadjusted and non-randomised. Embase, Web of Science and Scopus were not searched; although olorofim is an unambiguous search term and the journal literature that these databases index beyond MEDLINE is covered by Crossref and Europe PMC, reports indexed only in one of them cannot be excluded with certainty. Screening, extraction and appraisal were performed by a single reviewer; full-text searching of Europe PMC, Google Scholar and similar indexes was limited by access restrictions; and conference abstracts and the phase 3 press release were excluded by design. So, recent unpublished data and case reports mentioning olorofim only in the body text may have been missed, although the catalogued abstracts were checked against the included reports and the web queries were designed to retrieve body-text mentions.

## 5. Conclusions

The published clinical evidence for olorofim is recent, uncontrolled and partly overlapping, but it consistently shows that oral olorofim can produce a strict adjudicated response in a minority of heavily pretreated patients, stabilise or improve disease in most, control disseminated coccidioidomycosis in a large proportion of patients, and cure localised *Lomentospora*, *Scedosporium* and *Microascus* infections when combined with surgical source control. Treatment failures cluster in disseminated, endovascular and central nervous system *Lomentospora* disease and in refractory coccidioidal meningitis. Hepatic enzyme elevation is the principal adverse effect and usually responds to dose modification, although permanent cessation is occasionally required. Olorofim is therefore a reasonable salvage option for resistant mould and *Coccidioides* infections within trials or access programmes, while its relative efficacy, optimal duration, role in combination therapy and long-term safety await randomised trials and prospective registries.

## Figures and Tables

**Table 1:** Characteristics of the included studies.

Author	Year	Ref.	Design	Country	Olorofim, *n*	Other groups, *n*	Age (Years)	Immune Status
Maertens	2025	[17]	Phase 2b single-arm trial	Multinational (11)	203 (202 mITT)	None	Mean 52.9 (18–85)	Mixed (81 high, 62 moderate, 59 low/none)
Maertens	2026	[19]	Post hoc trial analysis	Multinational (11)	202	None	Mean 52.9 (18–85)	Mixed (141/161 non-coccidioidal IC)
Donovan	2026	[18]	Trial subgroup report	USA	41	None	Median 45 (21–72)	NIC 39/41
Thompson	2025	[20]	Nested case series	Multinational	6 (of 470)	None	Median 44 (29–74)	Mixed (4/6 NIC)
Esnault	2025	[21]	Retrospective cohort	France	17	None	Median 39 (14–73)	Mixed (15 IC)
Sastre-Escolà	2026	[62]	Retrospective cohort	Australia	5	13 (no olorofim)	Median 63.5 (IQR 56–68), cohort	IC
Faure	2023	[22]	Case series	France	3	None	17; 61; 65	Mixed (2 IC)
Michel	2023	[63]	Case report	France	1	None	14	IC
Dong	2024	[24]	Case report	Australia	1	None	57	IC
Febles Leyva	2024	[64]	Case report	Spain	1	None	73	NIC
Fortini	2025	[23]	Case report	USA	1	None	8	NIC
Chauhan	2025	[65]	Case report	USA	1	None	35	IC
van Mourik	2025	[58]	Case report	Netherlands	1	1 (no olorofim)	41	NIC
Carpenter	2026	[66]	Case report	USA	1	None	39	NIC
Ranade	2026	[30]	Case report	USA	1	None	67	IC
O’Donnell	2026	[67]	Case report	Australia	1	None	65	IC
Le Cœur	2026	[68]	Case report	France	1	None	73	IC
Hand	2026	[69]	Case report	Australia	1	None	30s	IC
Dunstan	2026	[31]	Case report	Australia	1	None	70s	IC
Cooper	2026	[70]	Case report	USA	1	None	73	IC
Mehta	2026	[71]	Case report	USA	1	None	NR (adult)	NIC

mITT, modified intention-to-treat population; IC, immunocompromised; NIC, non-immunocompromised; NR, not reported. Maertens 2026, Donovan 2026 and Thompson 2025 re-analyse patients of the phase 2b trial; Faure 2023 patients are included in Esnault 2025; Michel 2023 was treated within the same French programme.

**Table 2:** Pathogens, indications and olorofim treatment characteristics.

Study	Pathogen (*n*)	Site	Prior Failure or Intolerance	Olorofim MIC (mg/L)	Dose, Loading/Maintenance	Duration	Combined Antifungal	Surgery
Maertens 2025 [17]	*Aspergillus* 101 (22 azole-resistant); *L. prolificans* 26; *Scedosporium* 22; *Coccidioides* 41; other 12	Lung/sinonasal 125; CNS 48; bone/joint 34; disseminated 50	Failure 110; resistance 50; intolerance 29; other 13	NR	150 mg × 2 on day 1/90 mg bid (weight-based in first 58)	Median 84 d (2–99); extended median 309 d (*n* = 114)	≥15 d: 31/41 coccidioidal, 21/161 other; azole ≥2 d 64/202	NR
Maertens 2026 [19]	*Aspergillus* 101; *Coccidioides* 41; other 60	Multiple (NR)	Yes (202/202)	NR	150 mg × 2 on day 1/90 mg bid (weight-based in first 58)	Median 84 d (2–99); extended median 309 d (*n* = 114)	Azole 24/101; 30/41; 10/60	NR
Donovan 2026 [18]	*Coccidioides* 41	CNS 30 (devices 13); bone/soft tissue 8; other 3	Yes (41/41; mean 3.5 prior antifungals)	NR	150 mg × 2 on day 1/90 mg bid; 60 mg bid with CYP inhibitors (weight-based in first 4)	Mean 382 d; median 409 d (IQR 182–523; range 38–931)	34/41 (azole 21; azole + L-AmB 9; L-AmB 4)	NR
Thompson 2025 [20]	*Coccidioides* 3; *S. apiospermum*/*Scedosporium* 2; *L. prolificans* 1	Lung 1; CNS 2; mycetoma 1; disseminated 1; sinus 1	Yes (6/6; prior therapy median 216 d)	NR	150 mg × 2 on day 1/90 mg bid (trial regimen)	NR (reaction at median 41 d, range 6–253)	NR	NR
Esnault 2025 [21]	*Aspergillus* 14 strains; *Scedosporium* 3; *Microascus* 3; *L. prolificans* 1; *Rasamsonia* 1; other 1	Lung 15/17; CNS 4/17; disseminated 5/17	Yes (17/17)	0.007–0.5 (*n* = 8)	150 mg bid day 1/90 mg bid	Responders median 9 mo (IQR 3.5–12)	14/17	5/17 (before olorofim)
Sastre-Escolà 2026 [62]	*L. prolificans*, *Scedosporium* (*n* = 5)	Rhino-sino-orbital/CNS	Yes (progression 3; intolerance 1; subtherapeutic voriconazole 1)	0.125–0.5 (*n* = 5)	NR	NR (cohort median 197 d)	1/5 (voriconazole + terbinafine); monotherapy 4/5	NR (cohort 9/18)
Faure 2023 [22]	*Microascus* 3	Lung 3/3	Yes (3/3)	NR	180 mg bid day 1/90 mg bid (*n* = 2); 90 mg bid (*n* = 1)	6 wk; ≥9 mo; ≥3 mo	2/3 (terbinafine)	0/3
Michel 2023 [63]	*A. fumigatus* TR34/L98H 1	Lung, chest wall	Yes (voriconazole, L-AmB nephrotoxicity)	0.03	NR/3 mg/kg/day (troughs 1.24 and 1.22 mg/L)	≥2 y	Yes (caspofungin, initially)	Yes (lobectomy)
Dong 2024 [24]	*L. prolificans* 1	Eye, blood, aortic valve	Yes	NR	150 mg/90 mg bid	≈3 mo (to death)	Yes (voriconazole, terbinafine)	Yes (vitrectomy)
Febles Leyva 2024 [64]	*S. apiospermum* 1	Knee	Yes	Not tested	60 mg bid, then 90 mg bid	6 mo	Yes (voriconazole, 3 mo)	Yes (3 debridements)
Fortini 2025 [23]	*L. prolificans* 1	Knee, patella	Yes	0.06	60 mg bid day 1/30 mg bid	6 mo	No	Yes
Chauhan 2025 [65]	*S. apiospermum* 1	Lung	Yes	NR	NR	≥12 mo	Yes (initial 3 mo)	Yes (redo lung transplant)
van Mourik 2025 [58]	*Scedosporium*, *A. fumigatus* 1	Lung, pleura	Yes	NR	NR	NR	Yes (voriconazole, later)	No (pleural drainage)
Carpenter 2026 [66]	*L. prolificans* 1	Knee	Yes	0.125	150 mg bid day 1/90 mg bid	6 mo	No (local spacer)	Yes (9 debridements)
Ranade 2026 [30]	*Coccidioides* 1	Lung, CNS	Yes	NR	NR/90 mg bid; rechallenge 30 mg bid	4 wk; then 8 wk	No	No
O’Donnell 2026 [67]	*L. prolificans* 1	Elbow	Yes	0.25	NR/90 mg bid	24 wk	No	Yes (amputation)
Le Cœur 2026 [68]	*Scedosporium* sp. 1	Blood, knee	Yes	0.015	NR/90 mg bid	9 mo	No	Yes
Hand 2026 [69]	*L. prolificans* 1	Mitral valve, CNS, blood	No (added day 7)	0.125	150 mg bid day 1/90–150 mg bid	69 d	Yes	Yes (valve surgery ×2)
Dunstan 2026 [31]	*L. prolificans* 1	Muscle abscesses	Yes (intolerance)	NR	NR/90 mg bid	4 mo	No	No
Cooper 2026 [70]	*L. prolificans* 1	Lung, eye, CNS, spleen	Yes	0.06	150 mg bid (enteral)	3 d	No	No
Mehta 2026 [71]	*Coccidioides* 1	CNS	Yes	NR	NR/90 mg bid	11 mo	Yes (fluconazole, initially)	No

MIC, minimum inhibitory concentration (EUCAST or CLSI, as reported); bid, twice daily; CNS, central nervous system; NR, not reported. Trial dosing: 150 mg twice on day 1 then 90 mg twice daily from patient 59 onwards; the first 58 patients received a weight-based regimen (180 to 300 mg on day 1, then 120 to 240 mg/day).

**Table 3:** Efficacy, mortality, adverse events and follow-up in the included studies.

Study	Outcome Measure	Response, *n*/*N* (%)	Mortality, *n*/*N* (%)	Relapse	Hepatic AE, *n*/*N* (%)	Stopped for AE, *n*/*N*	Follow-Up
Maertens 2025 [17]	DRC global response, day 42/84	58/202 (28.7)/55/202 (27.2); with stable disease 152/202 (75.2)/128/202 (63.4). Day 42 by pathogen: *Aspergillus* 35/101; *L. prolificans* 11/26; *Scedosporium* 8/22; other 4/12; *Coccidioides* 0/41	24/202 (11.9) day 42; 33/202 (16.3) day 84 (*Aspergillus* 26/101; *L. prolificans* 3/26; *Scedosporium* 3/22; other 1/12; *Coccidioides* 0/41); 50/203 (24.6) whole study	NR	20/203 (9.9); 5 serious	6/203	Day 84; extension up to 988 d
Maertens 2026 [19]	DRC global response, aspergillosis, day 42/84	Azole 11/24 (45.8)/9/24 (37.5); no azole 24/77 (31.2)/25/77 (32.5)	Aspergillosis day 84: azole 7/24 (29.2); no azole 19/77 (24.7)	NR	Azole 2/34, 6/30; no azole 9/127, 3/11	NR	Day 84
Donovan 2026 [18]	DRC clinical success, day 42/84; investigator-assessed in extension	31/41 (75.6)/30/41 (73.2); day 180 26/38 (68.4); last visit 24/39 (61.5)	0/41 (0) day 84; 4/41 (9.8) end of extension (3 DCM)	Progression 6/39 at last visit	9/41 (22.0); GI related 1/41	1/41	Median 430 d (144–961)
Thompson 2025 [20]	Paradoxical reaction resolution	6/6 (100) resolved (7–87 d) and responded; 1 brief pause	0/6	NR	NR	0/6	NR
Esnault 2025 [21]	EORTC/MSG control (≥15 d treated)	Success 5/15 (33.3); partial 7/15 (46.7); failure 3/15 (20.0)	5/17 (29.4) at 3 mo	1/5 after stopping	1/17 (5.9)	0/17	To Dec. 2024
Sastre-Escolà 2026 [62]	Survival (cohort)	Alive at 30 d 4/5; alive beyond 180 d 3/5	Cohort 10/18 (56) at 30 d, 12/18 (67) at 180 d	NR	NR	NR	180 d
Faure 2023 [22]	Investigator assessed	3/3 (100) improved	1/3 (33), non-fungal	0/3	0/3	0/3	3–12 mo
Michel 2023 [63]	Investigator assessed (MRI)	1/1 complete remission	0/1	0/1	0/1	0/1	2 y
Dong 2024 [24]	Investigator assessed	0/1	1/1 (116 d)	1/1	0/1	0/1	116 d
Febles Leyva 2024 [64]	Investigator assessed	1/1 cure	0/1	0/1	0/1	0/1	≥6 mo
Fortini 2025 [23]	Investigator assessed	1/1 cure	0/1	0/1	0/1	0/1	24 mo
Chauhan 2025 [65]	Investigator assessed	1/1 culture clearance	0/1	0/1	NR	0/1	12 mo
van Mourik 2025 [58]	Investigator assessed	1/1 haemoptysis resolved	0/1	NR	NR	0/1	NR
Carpenter 2026 [66]	Investigator assessed	1/1 cure	0/1	0/1	0/1	0/1	≈12 mo
Ranade 2026 [30]	RUCAM causality	NR	0/1	NR	1/1 (ALT 1642 U/L)	1/1 (rechallenge tolerated)	≈9 mo
O’Donnell 2026 [67]	Investigator assessed	0/1 medical failure; cure after amputation	0/1	0/1	NR	0/1	12 mo
Le Cœur 2026 [68]	Investigator assessed	0/1	0/1	NA	NR	0/1	≥4 mo
Hand 2026 [69]	Investigator assessed	0/1 (transient control)	1/1 (76 d)	1/1	0/1	0/1	76 d
Dunstan 2026 [31]	PET resolution	1/1	0/1	0/1	1/1 (ALT 1630 U/L)	1/1 (permanent)	NR
Cooper 2026 [70]	Investigator assessed	Not assessable	1/1 (3 d)	NA	NR	0/1	3 d
Mehta 2026 [71]	CSF antigen, symptoms	0/1	0/1	NA	1/1 (DDI hepatitis)	0/1	≥3 y

DRC, data review committee; AE, adverse event; ALT, alanine aminotransferase; DDI, drug–drug interaction; RUCAM, Roussel Uclaf Causality Assessment Method; PET, positron emission tomography; CSF, cerebrospinal fluid; NR, not reported; NA, not applicable. Hepatic AE, liver enzyme elevation at least possibly attributed to olorofim. Percentages are those reported by the authors. NR in the hepatic AE column indicates that the report did not address adverse events; adverse events were not addressed in 6 of the 14 single-patient reports, so the absence of a reported hepatic event in a case report does not establish that none occurred, and adverse-event counts from the case literature should be read as minimum values subject to safety reporting bias.

## Data Availability

All data analysed in this review were extracted from published reports cited in the reference list. The study-level data extraction records described in Section 2.4 are available from the corresponding author on request.

## Supplementary Materials

Supplementary Materials: The following supporting information can be downloaded at: https://www.mdpi.com/article/10.3390/pathogens15101041/s1, File S1. PRISMA 2020 checklist.

## Abbreviations

The following abbreviations are used in this manuscript:

AE	adverse event
ALT	alanine aminotransferase
CI	confidence interval
CNS	central nervous system
CSF	cerebrospinal fluid
CYP	cytochrome P450
DHODH	dihydroorotate dehydrogenase
DRC	data review committee
EORTC/MSGERC	European Organisation for Research and Treatment of Cancer/Mycoses Study Group Education and Research Consortium
IFD	invasive fungal disease
JBI	Joanna Briggs Institute
MIC	minimum inhibitory concentration
mITT	modified intention-to-treat
NR	not reported
OR	odds ratio
OSF	Open Science Framework
PET	positron emission tomography
PRISMA	Preferred Reporting Items for Systematic Reviews and Meta-Analyses
SWiM	Synthesis Without Meta-analysis

## Appendix A

Table A1 lists the 52 structured web queries used to supplement the database searches (Section 2.3). Each query combined “olorofim” with one term, in the form “olorofim” AND <term>, and was run manually in Google Search and in Google Scholar, with the first 50 results of each query screened by title and snippet. Pathogen and site terms were also run as exact phrases. The queries were repeated on 1 September 2026, the closing date of the search.

**Table A1:** Structured web queries used to supplement the database searches.

Term Category (Number of Queries)	Terms Combined with “Olorofim”
Pathogen names (12)	*Lomentospora prolificans*; *Scedosporium*; *Scedosporium apiospermum*; *Scedosporium boydii*; azole-resistant *Aspergillus fumigatus*; *Coccidioides*; coccidioidomycosis; *Microascus*; *Scopulariopsis*; *Rasamsonia*; *Fusarium*; *Talaromyces*
Access programmes (6)	compassionate use; expanded access; managed access; named patient; emergency investigational new drug; early access programme
Sites of infection (12)	osteomyelitis; septic arthritis; endocarditis; endophthalmitis; keratitis; meningitis; brain abscess; sinusitis; pneumonia; mycetoma; disseminated infection; cystic fibrosis
Outcome and safety terms (10)	case report; hepatotoxicity; drug-induced liver injury; transaminitis; death; cure; relapse; paradoxical reaction; therapeutic drug monitoring; drug interaction
Journal and conference names (12)	Clinical Infectious Diseases; Open Forum Infectious Diseases; Journal of Fungi; Mycoses; Medical Mycology Case Reports; Journal of Antimicrobial Chemotherapy; Antimicrobial Agents and Chemotherapy; Transplant Infectious Disease; Pediatric Infectious Disease Journal; ESCMID Global (ECCMID); IDWeek; Trends in Medical Mycology (TIMM)

ECCMID, European Congress of Clinical Microbiology and Infectious Diseases; TIMM, Trends in Medical Mycology. Terms are listed as entered; pathogen names were entered without italics.
