# Supplementary material for: Olorofim for Invasive Fungal Diseases in Patients with Limited or No Treatment Options: A Systematic Review
Source: Pathogens. 2026 Oct 1;15(10):1041. doi: 10.3390/pathogens15101041 (PMC13708700; doi:10.3390/pathogens15101041)
Supplement: Supplementary file 1 [file pathogens-15-01041-s001.zip › pathogens-4574208-supplementary.pdf]

PRISMA

PRISMA 2020 Checklist

File S1 PRISMA 2020 checklist for pathogens 4574208

Locations refer to the published article, Pathogens 2026, 15, 1041 (doi: 10.3390/pathogens15101041). Partial or unreported items are identified explicitly.

Section and Topic	Item #	Checklist item	Location where item is reported
**TITLE**			
Title	1	Identify the report as a systematic review.	Title page, p. 1: identified as a systematic review.
**ABSTRACT**			
Abstract	2	See the PRISMA 2020 for Abstracts checklist.	Abstract, p. 1. OSF is named; the registration identifier and review funding are not included. Limitations are briefly stated.
**INTRODUCTION**			
Rationale	3	Describe the rationale for the review in the context of existing knowledge.	Section 1, Introduction, pp. 1–2.
Objectives	4	Provide an explicit statement of the objective(s) or question(s) the review addresses.	Section 1, final paragraph, p. 2 (review objective).
**METHODS**			
Eligibility criteria	5	Specify the inclusion and exclusion criteria for the review and how studies were grouped for the syntheses.	Sections 2.2, p. 3, and 2.5, pp. 5–6 (eligibility and synthesis groups).
Information sources	6	Specify all databases, registers, websites, organisations, reference lists and other sources searched or consulted to identify studies. Specify the date when each source was last searched or consulted.	Section 2.3, pp. 3–4; Appendix A, p. 21. Search coverage and web-query repetition through 1 September 2026 are stated; separate dates for every source are not given.
Search strategy	7	Present the full search strategies for all databases, registers and websites, including any filters and limits used.	Section 2.3, pp. 3–4; Appendix A, Table A1, p. 21 (52 web-query strings, query format and screening limits). Database and registry terms and eligibility limits are described.
Selection process	8	Specify the methods used to decide whether a study met the inclusion criteria of the review, including how many reviewers screened each record and each report retrieved, whether they worked independently, and if applicable, details of automation tools used in the process.	Sections 2.1, pp. 2–3, and 2.4, pp. 4–5; Limitations, p. 19. Single-reviewer screening with author re-checking; independent duplicate screening is not reported.
Data collection process	9	Specify the methods used to collect data from reports, including how many reviewers collected data from each report, whether they worked independently, any processes for obtaining or confirming data from study investigators, and if applicable, details of automation tools used in the process.	Sections 2.1, pp. 2–3, and 2.4, pp. 4–5; Limitations, p. 19. Structured single-reviewer extraction with author re-verification; investigator contact is not described.
Data items	10a	List and define all outcomes for which data were sought. Specify whether all results that were compatible with each outcome domain in each study were sought (e.g. for all measures, time points, analyses), and if not, the methods used to decide which results to collect.	Sections 2.2, p. 3; 2.4, pp. 4–5; and 2.5, pp. 5–6. Outcomes and time points are described; a rule for selecting every compatible result is not fully specified.
	10b	List and define all other variables for which data were sought (e.g. participant and intervention characteristics, funding sources). Describe any assumptions made about any missing or unclear information.	Section 2.4, pp. 4–5 (14 extraction domains; missing values recorded as NR and not estimated).
Study risk of bias assessment	11	Specify the methods used to assess risk of bias in the included studies, including details of the tool(s) used, how many reviewers assessed each study and whether they worked independently, and if applicable, details of automation tools used in the process.	Sections 2.1, p. 3, and 2.5, p. 5; Limitations, p. 19 (JBI tools, ratings and single-reviewer appraisal).
Effect measures	12	Specify for each outcome the effect measure(s) (e.g. risk ratio, mean difference) used in the synthesis or presentation of results.	Section 2.5, pp. 5–6 (proportions, odds ratios and confidence intervals).

PRISMA 2020 Checklist

Section and Topic	Item #	Checklist item	Location where item is reported
Synthesis methods	13a	Describe the processes used to decide which studies were eligible for each synthesis (e.g. tabulating the study intervention characteristics and comparing against the planned groups for each synthesis (item #5)).	Sections 2.2, p. 3; 2.4, pp. 4–5; and 2.5, pp. 5–6 (design/pathogen groups, overlap and eligibility for pooling).
	13b	Describe any methods required to prepare the data for presentation or synthesis, such as handling of missing summary statistics, or data conversions.	Sections 2.4, pp. 4–5, and 2.5, pp. 5–6 (missing values, denominators, subgroup counts and continuity correction).
	13c	Describe any methods used to tabulate or visually display results of individual studies and syntheses.	Section 2.5, pp. 5–6; Tables 1–3, pp. 7–13; Figures 2–4, pp. 14–16 and captions.
	13d	Describe any methods used to synthesize results and provide a rationale for the choice(s). If meta-analysis was performed, describe the model(s), method(s) to identify the presence and extent of statistical heterogeneity, and software package(s) used.	Section 2.5, pp. 5–6 (SWiM narrative synthesis; random-effects logit model, DerSimonian–Laird estimator, heterogeneity statistics and Python packages).
	13e	Describe any methods used to explore possible causes of heterogeneity among study results (e.g. subgroup analysis, meta-regression).	Section 2.5, pp. 5–6 (grouping by design/pathogen); Discussion, pp. 16–19. Formal meta-regression or subgroup tests of heterogeneity are not reported.
	13f	Describe any sensitivity analyses conducted to assess robustness of the synthesized results.	Section 2.5, p. 6: sensitivity analysis adds 13 non-overlapping individually reported patients as one aggregated group to the trial/cohort analyses.
Reporting bias assessment	14	Describe any methods used to assess risk of bias due to missing results in a synthesis (arising from reporting biases).	Section 2.5, pp. 5–6. Bias considered qualitatively; statistical publication-bias testing omitted because fewer than ten independent studies contribute per outcome.
Certainty assessment	15	Describe any methods used to assess certainty (or confidence) in the body of evidence for an outcome.	Section 2.5, p. 6. Certainty described qualitatively because comparative data are absent; no formal outcome-level grading method is reported.
**RESULTS**			
Study selection	16a	Describe the results of the search and selection process, from the number of records identified in the search to the number of studies included in the review, ideally using a flow diagram.	Section 3.1, pp. 6–7; Figure 1, p. 5 (PRISMA flow diagram).
	16b	Cite studies that might appear to meet the inclusion criteria, but which were excluded, and explain why they were excluded.	Section 3.1, pp. 6–7 (excluded reports, citations and reasons).
Study characteristics	17	Cite each included study and present its characteristics.	Sections 3.2–3.3 and Tables 1–2, pp. 7–11; reference list, pp. 22–25.
Risk of bias in studies	18	Present assessments of risk of bias for each included study.	Section 3.6, p. 15; Figure 4, p. 16 (JBI item-level assessment).
Results of individual studies	19	For all outcomes, present, for each study: (a) summary statistics for each group (where appropriate) and (b) an effect estimate and its precision (e.g. confidence/credible interval), ideally using structured tables or plots.	Section 3.4 and Table 3, pp. 11–13; Section 3.5 and Figures 2–3, pp. 14–15. Precision is provided for quantitative analyses, not every descriptive outcome.
Results of syntheses	20a	For each synthesis, briefly summarise the characteristics and risk of bias among contributing studies.	Sections 3.2–3.6, pp. 7–15; Tables 1–3, pp. 7–13; Figures 2–4, pp. 14–16.
	20b	Present results of all statistical syntheses conducted. If meta-analysis was done, present for each the summary estimate and its precision (e.g. confidence/credible interval) and measures of statistical heterogeneity. If comparing groups, describe the direction of the effect.	Section 3.5 and Figures 2–3, pp. 14–15 (pooled proportions, confidence intervals, heterogeneity and unadjusted odds ratios).

PRISMA

PRISMA 2020 Checklist

Section and Topic	Item #	Checklist item	Location where item is reported
	20c	Present results of all investigations of possible causes of heterogeneity among study results.	Sections 3.4–3.5, pp. 11–15; Discussion, pp. 16–19 (descriptive pathogen/site and azole differences). No formal investigation of between-study heterogeneity is reported.
	20d	Present results of all sensitivity analyses conducted to assess the robustness of the synthesized results.	Section 3.5, p. 14: adding the aggregated case-report group gives pooled hepatic events of 13.0% (95% CI 5.7–26.7; I^2 = 69%) and mortality of 18.0% (13.3–24.0; I^2 = 2%).
Reporting biases	21	Present assessments of risk of bias due to missing results (arising from reporting biases) for each synthesis assessed.	Section 3.5, p. 15; Discussion and Limitations, pp. 16–19. Qualitative publication/reporting-bias concerns; no formal synthesis-specific assessment.
Certainty of evidence	22	Present assessments of certainty (or confidence) in the body of evidence for each outcome assessed.	Discussion and Limitations, pp. 16–19. Qualitative limitations of confidence; formal certainty ratings by outcome are not presented.
**DISCUSSION**			
Discussion	23a	Provide a general interpretation of the results in the context of other evidence.	Section 4, Discussion, pp. 16–19.
	23b	Discuss any limitations of the evidence included in the review.	Section 4, including Limitations, pp. 18–19 (limitations of the included evidence).
	23c	Discuss any limitations of the review processes used.	Limitations, p. 19 (single reviewer, search restrictions and excluded publication types).
	23d	Discuss implications of the results for practice, policy, and future research.	Section 4, pp. 16–19; Section 5, Conclusions, pp. 19–20 (practice and future research).
**OTHER INFORMATION**			
Registration and protocol	24a	Provide registration information for the review, including register name and registration number, or state that the review was not registered.	Section 2.1, pp. 2–3: Open Science Framework registration, osf.io/p9h4b.
	24b	Indicate where the review protocol can be accessed, or state that a protocol was not prepared.	Section 2.1, pp. 2–3: protocol access through the OSF registration, https://osf.io/p9h4b.
	24c	Describe and explain any amendments to information provided at registration or in the protocol.	Not reported: no explicit statement of protocol amendments or their absence.
Support	25	Describe sources of financial or non-financial support for the review, and the role of the funders or sponsors in the review.	Section 2.1, p. 3; Funding statement, p. 20 (no external research funding; publication costs supported by UMFVBT). Funder role is not explicitly stated.
Competing interests	26	Declare any competing interests of review authors.	Conflicts of Interest statement, p. 20.
Availability of data, code and other materials	27	Report which of the following are publicly available and where they can be found: template data collection forms; data extracted from included studies; data used for all analyses; analytic code; any other materials used in the review.	Data Availability Statement, p. 20: published source reports are cited; extraction records are available on request. Search queries are in Appendix A, p. 21. Public access to extraction forms and analytic code is not specified.

From: Page MJ, McKenzie JE, Bossuyt PM, Boutron I, Hoffmann TC, Mulrow CD, et al. The PRISMA 2020 statement: an updated guideline for reporting systematic reviews. BMJ 2021;372:n71. doi: 10.1136/bmj.n71. This work is licensed under CC BY 4.0. To view a copy of this license, visit https://creativecommons.org/licenses/by/4.0/
